# Road traffic noise, noise sensitivity, noise annoyance, psychological and physical health and mortality

**DOI:** 10.1186/s12940-021-00720-3

**Published:** 2021-03-25

**Authors:** Stephen Stansfeld, Charlotte Clark, Melanie Smuk, John Gallacher, Wolfgang Babisch

**Affiliations:** 1 Centre for Psychiatry, Wolfson Institute of Preventive Medicine, Barts and the London School of Medicine, Queen Mary University of London, Charterhouse Square, London, EC1M 6BQ UK; 2 Present address: Population Health Research Institute, St George’s, University of London, Cranmer Terrace, London, SW17 0RE UK; 3 Present address: Medical Statistics Department, London School of Hygiene and Tropical Medicine, Keppel Street, Bloomsbury, London, WC1E 7HT UK; 4 Department of Psychiatry, Warneford Hospital, Oxford, OX3 7JX UK; 5 Umweltbundesamt, Institut für Wasser-Boden-und Lufthygiene, Corrensplatz 1, D-14195 Berlin, Germany; 6 Present address: Himbeersteig 37, 14129 Berlin, Germany

**Keywords:** Environmental noise, Mental health, Cohort study, Heart disease, Vulnerability, Stress, Noise sensitivity

## Abstract

**Background:**

Both physical and psychological health outcomes have been associated with exposure to environmental noise. Noise sensitivity could have the same moderating effect on physical and psychological health outcomes related to environmental noise exposure as on annoyance but this has been little tested.

**Methods:**

A cohort of 2398 men between 45 and 59 years, the longitudinal Caerphilly Collaborative Heart Disease study, was established in 1984/88 and followed into the mid-1990s. Road traffic noise maps were assessed at baseline. Psychological ill-health was measured in phase 2 in 1984/88, phase 3 (1989/93) and phase 4 (1993/7). Ischaemic heart disease was measured in clinic at baseline and through hospital records and records of deaths during follow up. We examined the longitudinal associations between road traffic noise and ischaemic heart disease morbidity and mortality using Cox Proportional Hazard Models and psychological ill-health using Logistic Regression; we also examined whether noise sensitivity and noise annoyance might moderate these associations. We also tested if noise sensitivity and noise annoyance were longitudinal predictors of ischaemic heart disease morbidity and mortality and psychological ill-health.

**Results:**

Road traffic noise was not associated with ischaemic heart disease morbidity or mortality. Neither noise sensitivity nor noise annoyance moderated the effects of road traffic noise on ischaemic heart disease morbidity or mortality. High noise sensitivity was associated with lower ischaemic heart disease mortality risk (HR = 0.74, 95%CI 0.57, 0.97). Road traffic noise was associated with Phase 4 psychological ill-health but only among those exposed to 56-60dBA (fully adjusted OR = 1.82 95%CI 1.07, 3.07). Noise sensitivity moderated the association of road traffic noise exposure with psychological ill-health. High noise sensitivity was associated longitudinally with psychological ill-health at phase 3 (OR = 1.85 95%CI 1.23, 2.78) and phase 4 (OR = 1.65 95%CI 1.09, 2.50). Noise annoyance predicted psychological ill-health at phase 4 (OR = 2.47 95%CI 1.00, 6.13).

**Conclusions:**

Noise sensitivity is a specific predictor of psychological ill-health and may be part of a wider construct of environmental susceptibility. Noise sensitivity may increase the risk of psychological ill-health when exposed to road traffic noise. Noise annoyance may be a mediator of the effects of road traffic noise on psychological ill-health.

**Supplementary Information:**

The online version contains supplementary material available at 10.1186/s12940-021-00720-3.

## Background

Studies have linked long term exposure to road traffic noise to increased hypertension risk, myocardial infarction, cardiovascular and stroke mortality [1–4]. There is accumulating evidence that transport noise is related to an increased risk of depression, hypertension, stroke, cardiovascular disease and mortality [3–8]. The stress hypothesis has been put forward as the most likely mechanism underlying the effects of environmental noise on health where chronic noise exposure of sufficient intensity leads to increased stress responses, hypertension, metabolic syndrome, diabetes mellitus and increased risk of cardiovascular disease [9].

This paper examines whether noise sensitivity and noise annoyance moderate the effects of road traffic noise on ill-health and whether they might have direct effects on both physical and psychological ill-health. Noise sensitivity, based on scales of self-report responses to a range of sounds, has been used to differentiate people with a strong dislike of noise from those who are indifferent to noise or who are not bothered at all by noise, so-called ‘imperturbables’ [10]. Does everyone respond physiologically to noise exposure in the same way – probably not? However, whether high self-reported noise sensitivity equates to high levels of physiological responsiveness to noise and subsequent greater susceptibility to disease than for those with low noise sensitivity is uncertain. Noise sensitivity has been associated with some indices of raised physiological response (e.g. tonic heart rate and defence/startle responses to noise in the laboratory) but the strengths of the associations between autonomic nervous system functioning and noise exposure tend to be weak and inconsistent [11].

There is general consistency across studies, that noise sensitivity has a moderating effect on another self-report variable, noise annoyance [12–14]. Annoyance expresses mild anger, partly as a result of noise interference into everyday activities, coupled feelings of invasion of privacy and lack of control. It is often seen as a state or immediate response to noise but people’s annoyance responses tend to be stable over time suggesting a personality-based consistency to responding. There has been controversy over whether high levels of annoyance might be a transitional stage on the pathway from noise exposure to disease [15].

Annoyance as a mediator presupposes that high levels of emotional response associated with annoyance may be an outward manifestation of underlying physiological arousal. There is some evidence of noise annoyance as a mediator between noise exposure and depression [16] and mental ill-health [17]. There is recent evidence that noise sensitivity may be a moderator of the effects of environmental noise on physical ill-health, for instance, cardiovascular outcomes [18, 19] and possibly more likely, psychological ill-health [20, 21].

Noise sensitivity has direct associations with ill-health, for instance, with the award of disability pensions in Finland [22] and with health-related quality of life [23]. From twin studies there is evidence of an underlying genetic basis to noise sensitivity which could be linked to susceptibility to ill-health [24]. Studies have repeatedly found largely cross sectional associations with both psychological ill-health [11, 19, 25] and personality traits such as neuroticism and trait anxiety [26–28]. Noise sensitivity is also associated to sensitivity to other environmental stimuli [29]. Could trait anxiety, or a similar concept, negative affectivity, be part of a unifying construct of fearfulness of the risks of the external world that underlies noise sensitivity and a range of environmental sensitivities? In longitudinal analyses, in a UK study of civil servants noise sensitivity predicted common mental disorder but not coronary heart disease or cardiovascular mortality except in certain subgroups, namely as a predictor of angina in lower employment grades in the UK civil service [30]. In the Caerphilly study, at earlier phases, road traffic noise was demonstrated to be related longitudinally to symptoms of anxiety but not to more general measures of common mental disorder including depression as well as anxiety [31]. In this paper, in the Caerphilly Collaborative Heart Disease Study, a longitudinal cohort study of men, we examine: 1) the longitudinal association between road traffic noise and ischaemic heart disease morbidity and mortality and 2) whether noise sensitivity and noise annoyance might moderate these associations; 3) the longitudinal association between road traffic noise and psychological ill-health and 4) whether noise sensitivity and noise annoyance might moderate these associations. We also tested if noise sensitivity, independently of noise exposure, was a longitudinal predictor of ischaemic heart disease morbidity and mortality and psychological ill-health and finally whether noise annoyance was an independent longitudinal predictor of ischaemic heart disease morbidity and mortality and psychological ill-health. Psychological ill-health as defined in this paper includes psychological distress, anxiety and depression identified by the General Health Questionnaire and often referred to as ‘common mental disorder’. Our hypotheses were: i) road traffic noise exposure at baseline (phase 2) will predict ischaemic heart disease morbidity and mortality at follow-up between phase 2 and phase 4; ii) noise sensitivity and noise annoyance will not moderate the association between traffic noise exposure and ischaemic heart disease; iii) road traffic noise exposure will not directly predict psychological ill-health; iv) noise sensitivity and noise annoyance will moderate the association of road traffic noise exposure on psychological ill-health; v) there will be no direct association of noise sensitivity with ischaemic heart disease or mortality; vi) there will be no direct association of noise annoyance with ischaemic heart disease or mortality; vii) that noise sensitivity will predict future psychological ill-health; viii) that noise annoyance will predict future psychological ill-health. An earlier version of this paper was published as a conference paper at the International Congress on Noise as a Biological Health Problem in 2017 [32].

## Methods

### Sample

The Caerphilly Collaborative Heart Disease Study [33] was set up as a cohort study of men in South Wales in the early 1980’s to investigate risk factors for ischaemic heart disease (IHD). Men 45–59 years old living in Caerphilly, South Wales, UK, and surrounding villages were eligible for inclusion. Initial screening included self-report questionnaires and clinic visits for anthropometry, blood pressure measurement and blood samples for cardiac risk factors. Initially, at Phase 1, (1979–83), 2512 (89%) of the eligible 2818 men were screened [34] (Table 1). At phase 2 (1984–88), the first follow-up the cohort was enhanced by 447 additional men who had either recently moved to the area or who had been missed at Phase 1. By Phase 2, 561 men had been lost to the cohort from the Phase 1 sample. This established a new cohort baseline for the 1984/88 population-based study comprising of 2398 men. At phase 3, (1989–93) 2154 men were seen in the clinic. At phase 4, (1993–97)1701 men were seen in the clinic, 344 had died and 353 had either moved or refused to take part.

**Table 1:** Screening phases of the Caerphilly study: Phases 1 to 4

Screening Phase	Years	N	Comment
Phase 1	1979–83	2512/2818	Response rate 89%
Phase 2	1984–88	2398	477 added, 561 lost
Phase 3	1989–93	2154	
Phase 4	1993–97	1701	344 died, 353 moved

### Traffic noise exposure

In 1984 measurement of A-weighted sound pressure level was carried out street by street to derive maps of road traffic noise [35]. On three consecutive days noise measurements were carried out continuously involving all busy roads and many side streets. Additionally, short-term measurements of L_eq 30 min_ were conducted during representative periods of the day (10.00–18.00 h) on all other relevant streets. Most traffic exposed dwellings were within 12 m from the street. Using the noise measurements and the maps derived therefrom participants were categorised into 5 dB groups of traffic noise emission level, in terms of Leq referring to the period from 6.00 to 22.00 and at a distance of 10 m from the street. Daytime outdoor noise level was then used as a general metric of street traffic noise. Due to the architecture of the housing, largely terraced houses, traffic noise emissions and emission level (perceived at the facades) were very similar for the vast majority of men. No major changes in noise level were found between phases 2 and 3 of the study [36]. More sophisticated mapping was not available in the 1980s.

### Noise sensitivity

Weinstein’s 10-item self-report noise sensitivity scale, derived from his original 21 item scale, was used to measure noise sensitivity at Phase 2 baseline [29]. Scores were divided into equal tertiles of low (<=20), medium (21–27), and high (> = 28) sensitivity for analysis. Cronbach’s alpha for this scale in the preliminary sample at baseline was 0.78 [28].

### Noise annoyance

Self-reported road traffic noise annoyance was measured by a single question administered at Phase 2 baseline: ‘Does traffic noise at home annoy you?’ with five ordered levels of response from ‘never’ to ‘always’. For the purpose of analysis ‘never’, ‘seldom’ and ‘sometimes’ were classified as low annoyance and ‘often’ and ‘always’ as high annoyance.

### Ischaemic heart disease morbidity and mortality

Electrocardiogram (ECG) and cardiac enzyme levels were used to identify possible ischaemic heart disease events. These were obtained from hospital records and were evaluated against standard diagnostic criteria; Incident ischaemic heart disease (IHD) events were defined as: IHD death (ICD-9 codes 410–414); non-fatal myocardial infarction (MI) (a cardiac event satisfying WHO criteria); and electrocardiographic evidence of MI (major or moderate Q/QS waves, Minnesota codes 1–1-1 to 1–2-5 or 1–2-7 on any follow up ECG when there were no Q/QS waves, Minnesota codes 1–1-any, 1–2-any, or 1–3-any on the recruitment ECG) [34]. Information on deaths was obtained from notifications to the Office of National Statistics.

### Psychological ill-health

The 30-item General Health Questionnaire (GHQ), which measures common mental disorder, predominantly depression and anxiety, was used to identify psychological ill-health [37]. A validity study was carried out using the Clinical Interview Schedule in a subsample of 97 men from the study. A consecutive sample of clinic attenders stratified by GHQ score to provide one third high scorers, and two thirds low scorers was selected by another team member from the first 1100 clinic attenders over 18 months. A threshold of 4/5 on the GHQ was established distinguishing between ‘probable non-cases’ and ‘probable cases’ [38]. Measurements of psychological ill-health were taken at phase 2 baseline in 1984/88, at phase 3 follow up 1989/93 and phase 4 follow up in 1993/7.

### Covariates

At baseline, smoking history, alcohol history, physical activity at leisure, previous history of cardiovascular disease, noise at work and Registrar General classification of social class were obtained by questionnaire. The British Registrar General classification is an occupation-based classification of social class that includes the following categories: ‘I and II’ professional/managerial/technical, ‘IIINM’ other non-manual, ‘IIIM’ skilled manual, and ‘IV and V’ unskilled manual. These categories may be merged into ‘non-manual’, (I, II, IIINM) and ‘manual’ occupations (IIIM, IV, and V). BMI was calculated after height was measured on a Holtain stadiometer and body weight using a beam balance.

### Statistical analysis

Cox Proportional Hazard Models were used to analyze the association of road traffic noise, noise annoyance and noise sensitivity with IHD morbidity and mortality. The models for each predictor and outcome were initially run univariately and then run adjusted for age, social class, marital status, and employment status, smoking status, BMI, alcohol consumption, physical activity at leisure, and noise at work. For the road traffic noise models, additional individual adjustments were made to the adjusted models firstly, for noise annoyance and secondly, for noise sensitivity to see if these factors separately mediated the associations between noise exposure and IHD morbidity and mortality. Interactions between noise exposure and noise annoyance, and noise exposure and noise sensitivity, were also examined to see if these factors moderated the associations between noise exposure and IHD morbidity and mortality. Cox proportional hazard models were tested using Schoenfeld residuals, with the assumptions being met for the adjusted models for both IHD morbidity and mortality.

Logistic regression was used to analyze the association of road traffic noise, noise annoyance and noise sensitivity with psychological ill-health. The models for each predictor and outcome were initially run univariately and then run adjusted for age, social class, marital status, employment status, smoking status, BMI, alcohol consumption, physical activity at leisure, and noise at work. Additional individual adjustments to the model for noise exposure were then made firstly, for noise annoyance and secondly, for noise sensitivity to see if these factors mediated the associations between noise exposure and psychological ill-health. Interactions between noise exposure and noise annoyance, and noise exposure and noise sensitivity, were also examined to see if these factors moderated the associations between noise exposure and psychological ill-health.

Stata Version 14 (StataCorp, 2015) was used to perform all data analysis and all analyses were assessed at the 5% statistical level to define statistical significance. Results are presented using coefplot [39].

### Missing data

The sample size of the 1984/88 (phase 2) population-based cohort comprised of 2398 men. The analyses represented in this paper are based on complete-case analyses per outcome, due to the limitation of predictive variables to impute missing observations. The sample for the IHD mortality and morbidity analyses at phase 2 was therefore reduced to *n* = 1868 as item responses ranged from 0 to 13.6% (cholesterol). The sample sizes for the logistic regression models based on phase 3 (*n* = 1512) and phase 4 (*n* = 1320) data were reduced considerably as the GHQ was poorly completed (27.9% missing at phase 3 and 37.7% missing at phase 4).

## Results

### Descriptives

Sociodemographic characteristics of the participants and key data relating to noise exposure, noise annoyance, noise sensitivity, IHD mortality and morbidity and psychological ill-health are reported in Table 2. A supplementary table provides odds ratios of the main exposures and outcomes.

**Table 2:** Sociodemographic characteristics of the study sample

	N (2398 at baseline) or mean (standard deviation)	Percentage
**Covariates**		
Age		
45–54 years	840	35.3
55–60 years	767	31.8
> 60 years	791	32.9
Marital Status		
Widowed, divorced, separated	177	7.4
Single	122	5.1
Married	2099	87.5
Registrar General Social Class		
Non-manual	780	32.6
Manual	1610	67.4
Employment status		
Employed	1107	43.6
Self-employed	152	6.3
Unemployed	362	15.1
Retired	770	32.3
Smoking status		
Non-smoker	431	18.0
Ex-smoker	907	37.9
Current smoker	1054	44.1
BMI	26.4 (3.64)	Not applicable
Alcohol consumption ccs/week	15,414 (199)	Not applicable
Physical activity	130,252 (134355)	Not applicable
Noise at work		
1	747	32.1
2	878	37.8
3	700	30.1
**Noise variables**		
Noise exposure (LAeq 16 h)		
51-55dBA	2101	72.6
56-60dBA	247	8.5
61-65dBA	374	12.9
66-70dBA	174	6.0
Noise sensitivity		
Lowest tertile	808	34.6
Mid tertile	773	33.1
Highest tertile	753	32.3
Noise annoyance		
Never, Seldom, Sometimes	2262	96.4
Often, Always	84	3.6
**Cardiovascular variables**		
CHD prior to phase 2		
No	2345	97.8
Yes	53	2.2
IHD mortality between phase 2 and phase 4		
No	1976	82.4
Yes	422	17.6
IHD morbidity between phase 2 and phase 4		
No	1659	69.2
Yes	739	30.8
**Psychological variables**		
GHQ2		
No	7177	77.7
Yes	490	22.3
GHQ3		
No	1319	76.2
Yes	410	23.8
GHQ4		
No	1124	75.3
Yes	369	24.7

### IHD mortality and morbidity

**Fig. 1:**
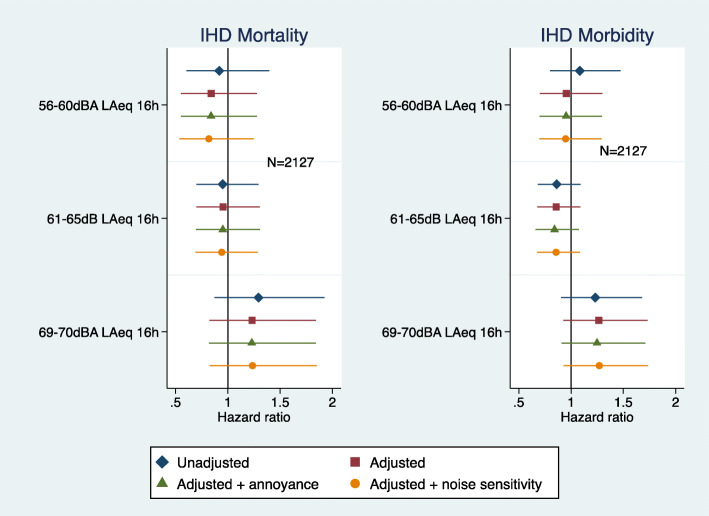
Road traffic noise exposure with IHD mortality and IHD Morbidity (adjusted = age, marital status, social class, employment, smoking status, BMI, alcohol consumption, physical activity at leisure, and noise at work)

Road traffic noise exposure was not associated with IHD mortality or morbidity (Fig. 1). The associations were not mediated with further adjustment for noise annoyance or noise sensitivity (Fig. 1). In the unadjusted model there was a suggestion of lower IHD morbidity in the 61–65 dBA noise category but this was not observed in the fully adjusted model.

**Fig. 2:**
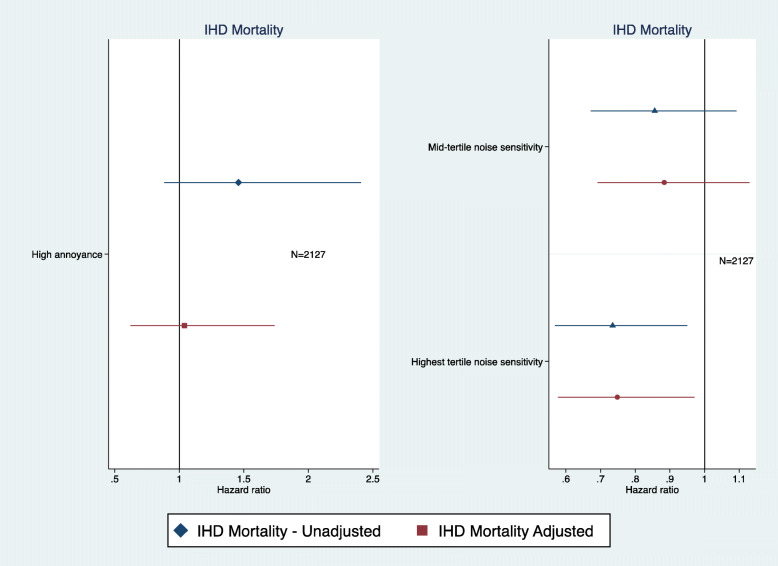
Road traffic noise annoyance and noise sensitivity associations with IHD mortality (adjusted = age, marital status, social class, employment, smoking status, BMI, alcohol consumption, physical activity at leisure, and noise at work)

In order to test the full impact of noise sensitivity on physical health, associations with IHD mortality were examined. IHD mortality rather than all-cause mortality was selected because of previous analyses showing associations between environmental noise and IHD mortality. High noise sensitivity, somewhat unexpectedly, was found to be associated with a lower risk of IHD mortality than medium and low noise sensitivity even after full adjustment (HR = 0.74, 95%CI 0.57–0.97) (Fig. 2). Noise annoyance was not associated with IHD mortality (Fig. 2).

**Fig. 3:**
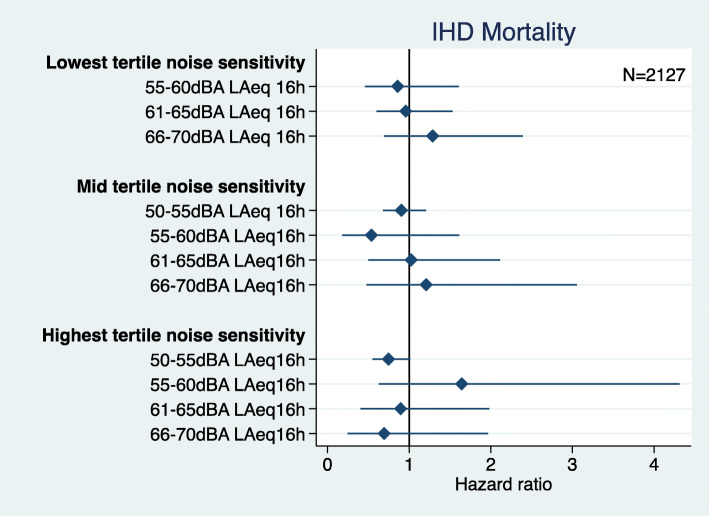
Road traffic noise interaction with noise sensitivity and IHD mortality (adjusted = age, marital status, social class, employment, smoking status, BMI, alcohol consumption, physical activity at leisure, and noise at work)

**Fig. 4:**
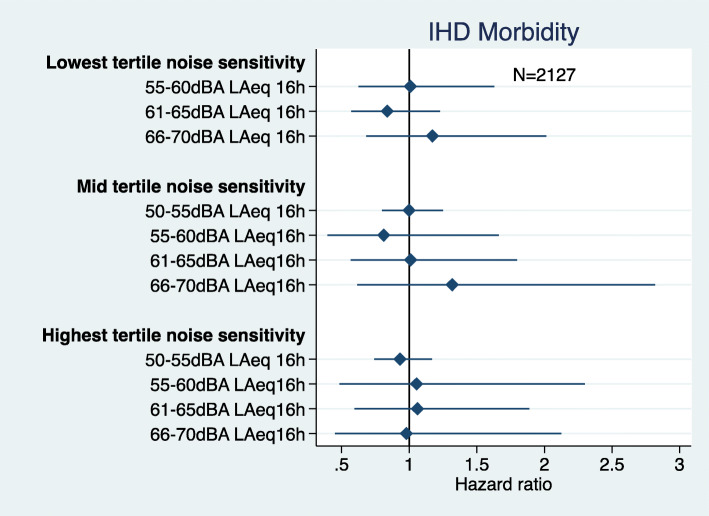
Road traffic noise interaction with noise sensitivity and IHD morbidity (adjusted = age, marital status, social class, employment, smoking status, BMI, alcohol consumption, physical activity at leisure, and noise at work)

There was no statistically significant interaction between road traffic noise exposure and noise sensitivity and IHD mortality (Fig. 3), however, the odds for being exposed to 50-55 dB and having high noise sensitivity was borderline significant (OR 0.74 95%CI 0.55, 1.01). There was no statistically significant interaction between road traffic noise exposure and noise sensitivity and IHD morbidity (Fig. 4). There was low power to examine the interaction between road traffic noise exposure and annoyance with either IHD mortality or morbidity, so this hypothesis was not further examined.

### Psychological ill-health

**Fig. 5:**
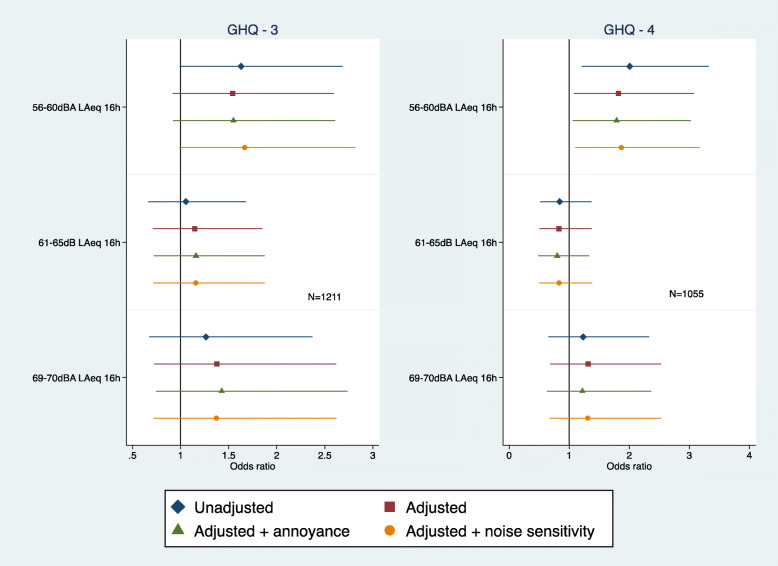
Road traffic noise and psychological ill-health at phase 3 and phase 4 (adjusted = age, marital status, social class and employment, smoking status, alcohol consumption, noise at work and physical activity at leisure)

When examining incident cases of psychological ill-health a sample was selected from which GHQ cases were removed at baseline (phase 3 sample *n* = 1211; phase 4 sample *n* = 1055). A borderline significant association between road traffic noise at baseline and Phase 3 psychological ill-health was found before adjustment but only among those exposed to 56-60dBA (OR =  1.62 95%CI 0.98, 2.68) (Fig. 5). This association was not maintained in the final model after full adjustment (OR = 1.54 95%CI 0.91, 2.59). A similar statistically significant association was observed for Phase 4 psychological ill-health (OR 2.00 95%CI 1.21, 3.32) and this remained statistically significant after full adjustment (OR 1.82 95%CI 1.07, 3.07) (Fig. 5). These associations were not altered with further adjustment for noise annoyance or noise sensitivity.

**Fig. 6:**
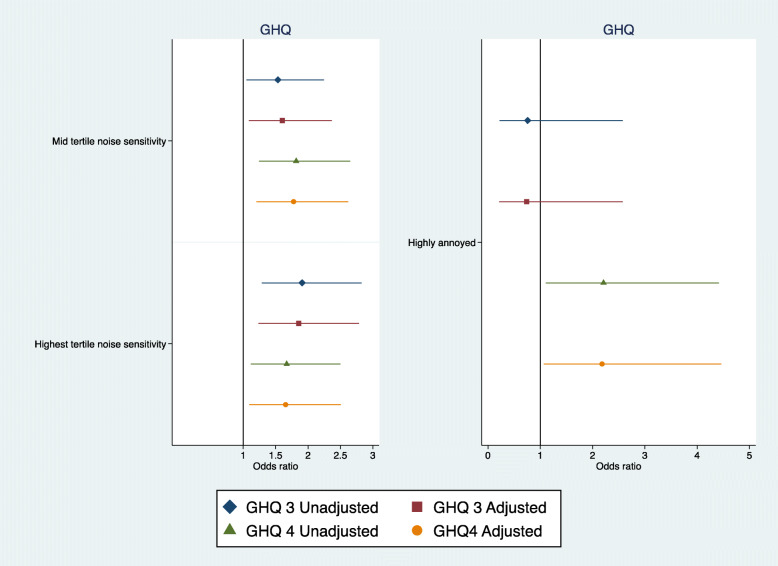
Road traffic noise annoyance and noise sensitivity associations with psychological ill-health at phase 3 and phase 4 (adjusted = age, marital status, social class and employment, smoking status, alcohol consumption, noise at work, and physical activity at leisure)

High and moderate levels of noise sensitivity at baseline were associated longitudinally with psychological ill-health at phase 3 even after full adjustment, (High noise sensitivity OR = 1.85 95%CI 1.23, 2.78; Moderate noise sensitivity OR = 1.60 95%CI 1.08, 2.36) (Fig. 6). Similarly, noise sensitivity predicted psychological ill-health at Phase 4 even after full adjustment (High noise sensitivity OR = 1.65 95%CI 1.09, 2.50; Moderate noise sensitivity OR = 1.77 95% CI 1.20, 2.62) (Fig. 6). High annoyance did not predict psychological ill-health at phase 3 but showed a borderline statistically significant association with psychological ill-health at phase 4 (Fully adjusted OR = 2.47 95%CI 1.00, 6.13).

**Fig. 7:**
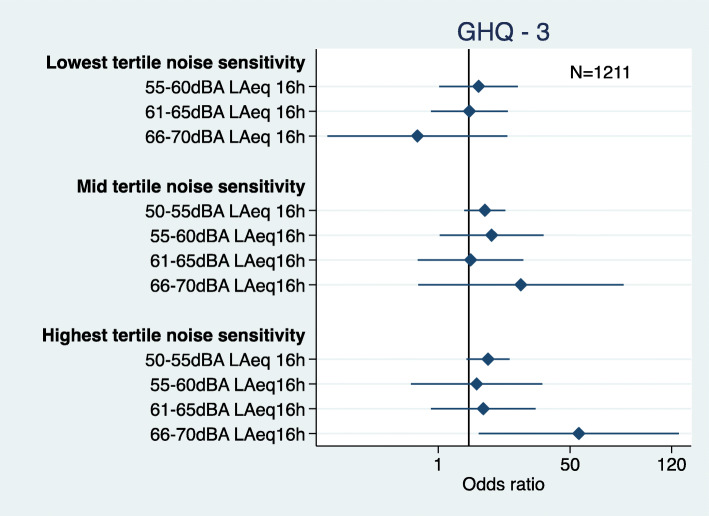
Road traffic noise and noise sensitivity interaction for psychological ill-health at phase 3 (adjusted = age, marital status, social class and employment, smoking status, alcohol consumption, noise at work, and physical activity at leisure)

There was an interaction between road traffic noise and noise sensitivity with phase 3 psychological ill-health (Fig. 7). The men who were highly noise sensitive in the 66-70dBA (highest) noise exposure group had a high odds of psychological distress (OR = 12.16 95%CI 1.25, 118.10). There was high variability around this estimate.

**Fig. 8:**
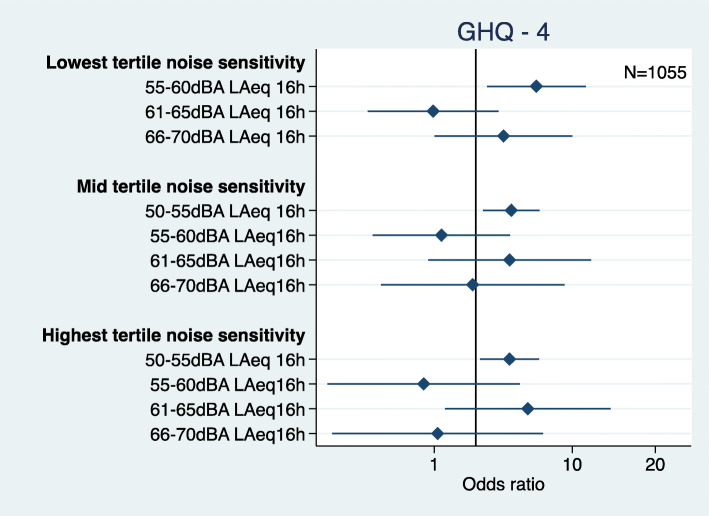
Road traffic noise and noise sensitivity interaction for psychological ill-health at phase 4 (adjusted = age, marital status, social class and employment, smoking status, alcohol consumption, noise at work, and physical activity at leisure)

There was also an interaction between road traffic noise and noise sensitivity with psychological ill-health at phase 4 (Fig. 8). High levels of noise sensitivity at baseline were associated with psychological ill-health at phase 4 for men in the 50-55 dB exposure group (OR 1.75 95%CI 1.07, 2.78). Moderate noise sensitivity was associated with psychological ill-health at phase 4 for men in the 50-55 dB exposure group (OR 1.79 95%CI 1.19, 2.68). However, odds for psychological health were also higher for men in the 55-60 dB exposure group with low noise sensitivity (OR 2.74 95%CI 1.20, 6.25).

There was low power to examine the interaction between road traffic noise exposure and annoyance with psychological ill-health at phase 3 and phase 4, so this hypothesis was not further examined.

## Discussion

Road traffic noise exposure was not associated longitudinally with IHD morbidity and mortality in these cohort analyses. As hypothesised the interaction of road traffic noise, noise sensitivity and IHD outcomes was not statistically significant. Earlier analyses in this cohort, at 10 year follow up, showed an increased relative risk of IHD in relation to road traffic noise especially among those living for 15 years or more in the same place [36]. Exposure misclassification and the lengthening of the interval between baseline traffic noise assessment and ascertainment of health outcomes may explain the lack of association in these recent analyses. There may have been self-selection out of the noisiest areas or not into noisy areas, even at baseline, for the most noise sensitive, or this could have occurred during follow up. However, mobility of noise sensitive persons out of noisy areas has not been found in other studies [40].

Previous studies have also found that noise sensitivity did not moderate the association of road traffic noise exposure and IHD events [16], although noise sensitivity has been found to moderate the effects of aircraft noise on hypertension [19]. Similarly, noise sensitivity was not a predictor of cardiovascular outcomes in an earlier study of civil servants except for participants in the lower employment grades where it predicted angina pectoris [30]. This is in contrast to a study of Finnish twins where noise sensitivity was a predictor of cardiovascular mortality in women but not in men [41]. Such a gender difference is in keeping with the results from the Caerphilly Study in men but not with the results in civil servants both men and women (Whitehall II Study), although in the latter study men and women were combined for analysis. A further explanation might lie in the outcomes chosen. In the Finnish Study noise sensitivity was a significant predictor of cardiovascular mortality (ICD codes 390–459,100–199) but not coronary heart disease mortality (ICD codes 410–414, 120–125). In the Caerphilly Study we only included ischaemic heart disease (also known as coronary heart disease ICD codes 410–414). Although this outcome measurement issue would not explain the lack of results in the Whitehall Study which did include cardiovascular outcomes such as stroke morbidity and mortality.

In our findings there was no support for annoyance being a moderating factor of the relationship between road traffic noise exposure and ischaemic heart disease [15]; effect modification of noise on hypertension was only demonstrated for aircraft noise and not road traffic noise in the HYENA study [15]. As there was no direct relationship between noise annoyance and IHD outcomes, annoyance was not supported as a mediating factor between noise exposure and IHD [42]. However, in previous analyses in this cohort, in samples containing men from both areas of Caerphilly and Speedwell, an association between annoyance and incident IHD was only found in those participants without pre-existing chronic disease, where it was surmised that the lack of association in those with pre-existing chronic disease might be due to recall bias [43]. In general, a single question on noise annoyance was possibly not a strong enough outcome measure to test this hypothesis and a frequency measure of noise annoyance is not the same as the standardised ICBEN degree of annoyance measure used in many noise studies. Moreover, the ICBEN measure was developed after this cohort study was initiated.

If noise sensitivity were an independent predictor of physical ill-health it should be associated increased mortality rates. We did not expect it to be associated with lower mortality rates, in keeping with a previous study [18]. Lee et al. [44] found that highly anxious young people had lower accident mortality up to the age of 25 years because they tended to avoid putting themselves in high risk situations which could have high mortality risk attached. Our cohort was middle-aged and older men, not strictly comparable with the population in Lee et al’s study, nevertheless, it may be that noise sensitive people are more cautious and less likely to take risks that could increase mortality. Noise sensitivity has been associated with phobic disorders in a sample of women [45] and fearfulness and avoidance which are part of phobic disorders might be associated with noise sensitivity and could be associated with health-protective behaviours. Earlier analyses in this cohort found an association between noise level and noise sensitivity with less highly sensitive men living in the highest noise exposure areas so it may be that more sensitive men tend to choose to live in less noisy areas where that choice is possible [28]. It does not seem that this effect on mortality is mediated through health behaviours as our results were adjusted for smoking, leisure-time physical activity, BMI and alcohol use. Lower mortality rates were also found when noise sensitivity was replaced by trait anxiety in the models [32] supporting an essential role for long-term anxiety in this association.

There was an inconsistent association between road traffic noise and psychological ill-health, although there may be insufficient power in these analyses. Associations between road traffic noise and depressive symptoms and insurance claims for depressive illness have been found in previous studies [8, 46]. Earlier analyses in this cohort did find a longitudinal association between road traffic noise and symptoms of anxiety [31] and a recent meta-analysis has found an association between transportation noise and anxiety [47]. Noise sensitivity may moderate the effects of road traffic noise on psychological ill-health [21, 48], although there was some inconsistency between phases 3 and 4 and the confidence intervals were wide so that our analyses may have been underpowered. Independently of road or aircraft noise exposure noise sensitivity has been shown to be strongly associated with a range of common mental disorders [28, 45, 48, 49]. Most of these earlier studies have been cross sectional; this study and others [23, 30] have confirmed that noise sensitivity is associated with psychological ill-health longitudinally. Noise annoyance has been found to predict depression in previous longitudinal studies [16, 50]. In this study there was an association between noise annoyance and psychological ill-health at phase 4 but not at phase 3.

Noise sensitivity has also been associated with neuroticism [26, 28, 45, 51, 52]. Neuroticism is a construct similar to trait anxiety and has links to negative affectivity, a tendency to report life experiences and perceptions negatively. Noise sensitivity does not seem to be just trait anxiety or neuroticism and Shepherd et al. [40] have found higher correlations with introversion/extraversion than with neuroticism [53]. An important issue is does noise sensitivity reflect a response of being sensitive to noise alone or is it part of sensitivity to a wider range of environmental stimuli (e.g. light, odour, touch) [53]? If it is part of a more generalised sensitivity this could be driven by underlying chronic anxiety.

Noise sensitivity has been linked to sensitivity to other aspects of the environment such as sensitivity to chemicals, electromagnetic fields, light and odours [49, 54, 55]. Multiple Chemical Sensitivity (MCS) is a condition purported to be related to exposure to low levels of environmental chemicals which for most people would not result in health effects. In one study 73 % of MCS also were noise intolerant [54]. Another name for this condition that suggests it covers a broader spectrum of exposures than just chemicals is ‘Idiopathic Environmental Intolerance’ (IEI) [56]. A strong overlap has been found between Idiopathic Environmental Intolerance and Somatoform Disorders; more than half of IEI cases could be classified as Somatoform Disorders [57]. There was longitudinal stability of these conditions over a year and baseline negative affectivity and somatosensory amplification (a tendency to focus and amplify symptoms) predicted these conditions at 1 year follow up [58]. A strong association has been found between Idiopathic Environmental Intolerance and mood, anxiety and somatoform disorders across the lifecourse [59] and equally between MCS and major depressive disorder, generalised anxiety disorder and severe psychological distress [60].

It has been proposed that this environmental sensitivity may relate to a hyper-responsive central nervous system with increased reactivity of the limbic system in the brain although no physiological evidence has been found to support this [61]. However, noise sensitivity is not always accompanied by other environmental sensitivities. Baliatsas et al., [49] found noise sensitivity associated with environmental sensitivity in 9–50% of highly noise sensitive people in their general practice community sample. Heinonen-Guzejev and colleagues found noise sensitivity could be distinguished from MCS on the basis of factor analysis in the Finnish twin cohort study [62]. Thus although noise sensitivity may be a symptom of IEI or even somatisation disorder in some cases it is not necessarily associated with other environmental sensitivities in all cases [40, 62]. To that extent noise sensitivity is not a single reified entity but may be a non-specific indicator of sensitivity to sounds alone or part of a wider IEI or psychiatric syndrome. Thus noise sensitivity might have multiple origins [40].

Noise sensitivity has been associated with uncomfortable loudness levels in laboratory studies but has not been associated with especially sensitive hearing thresholds [63, 64]. Thus it does not seem to be related to abnormalities in the peripheral auditory system. The associations with sympathetic nervous system activity may reflect associations with state or trait anxiety rather than being specific to noise sensitivity [11, 65]. A study using electro- and magnetoencephalography measuring mismatch negativity found that noise sensitivity categorised with the Weinstein scale was associated with altered sensory processing in the auditory cortex implying a central cortical origin for noise sensitivity [66]. This is a type of neurophysiological validation of a self-report noise sensitivity scale but it does not directly link these auditory processing characteristics to vulnerability to ill-health as might be expected if noise sensitivity is related to increased susceptibility to ill-health. Intriguing exploratory EEG studies suggest that there may be a deficit in sensory gating in noise sensitive subjects leading to sensory ‘overload’ [65]. Noise sensitivity has also been associated with larger grey matter volume in several brain areas: bilaterally in the temporal poles and the hippocampus, left sided Heschl’s sulcus and the right anterior insula. Some of these brain areas may have relevance to the processing of sound by the auditory cortex [67]. Further research in these disciplines may well be productive.

Noise annoyance predicted psychological health longitudinally. This may be evidence for noise annoyance being a mediator between road traffic noise and psychological ill-health. Equally, because existing psychological ill-health tends to lead to increasing annoyance it may be that this association is being driven by prior psychological ill-health which at the same time is independently predicting future psychological ill-health. People who are already ill tend to report being more highly annoyed by noise than people who are not ill [68, 69].

It may be difficult to generalise too far from these results as the population, although representative of the local area, was confined to middle-aged and older men living in a very specific geographical area. A strength of the study was the careful ascertainment of cardiac outcomes, the high response rate and follow up response longitudinally. The psychological ill-health outcomes would have been stronger had we had a standardised psychiatric interview instead of a questionnaire. Missing data for psychological ill-health outcomes in phase 3 and 4 was a limitation. It was a limitation that we did not have noise measurement data for phase 3 to phase 4 data collection.

## Conclusions

There is evidence that noise sensitivity is related to susceptibility to psychological ill-health, in relation to noise exposure. Also noise sensitivity is a longitudinal risk factor for psychological ill-health independent of noise exposure. Annoyance is a weak predictor of future psychological ill-health. On balance there is more evidence for noise sensitivity being a moderator of the association of road traffic noise and psychological ill-health, not a mediator of the relationship. Conversely, noise annoyance seems to be potentially a mediator of the association of road traffic noise and psychological ill-health and not a moderator.

There needs to be further understanding of the neurophysiological correlates of noise sensitivity before much more progress can be made in the associations of noise sensitivity with ill-health.

## Supplementary Information

**Additional file 1.**

## Data Availability

The datasets generated or analysed in this study are available through the Data Custodian Professor Yoav Ben Shlomo at Bristol Medical School: Population Health Sciences (https://www.bristol.ac.uk/population-health-sciences/projects/caerphilly/about).

## Abbreviations

IHD: Ischaemic Heart Disease
Leq: Equivalent continuous sound level in decibels
dB: Decibel
ECG: Electrocardiogram
MI: Myocardial infarction
GHQ: General Health Questionnaire
BMI: Body Mass Index
HR: Hazard Ratio
OR: Odds Ratio
ICD: International Classification of Diseases
MCS: Multiple Chemical Sensitivity
IEI: Idiopathic Environmental Intolerance
EEG: Electroencephalogram
