# Supplementary material for: Road traffic noise, noise sensitivity, noise annoyance, psychological and physical health and mortality
Source: Environ Health. 2021 Mar 25;20:32. doi: 10.1186/s12940-021-00720-3 (PMC7995714; doi:10.1186/s12940-021-00720-3)
Supplement: Supplementary file 1 — Additional file 1. [file 12940_2021_720_MOESM1_ESM.docx]

Supplementary Table: summary of hazard ratios and odds ratios and their 95%CI showing the unadjusted and adjusted associations between noise exposure and IHD mortality and morbidity, and psychological distress at phase 3 and phase 4.

| **Noise exposure**  **(L_Aeq 16h_)** | **IHD Mortality**  **(n=2127)** | **IHD Morbidity**  **(n=2127)** | **GHQ Phase 3**  **(n=1211)** | **GHQ Phase 4**  **(n=1055)** |
| --- | --- | --- | --- | --- |
|  | Hazard ratio (95%CI) | Hazard ratio (95%CI) | Odds ratio  (95%CI) | Odds ratio  (95%CI) |
| **Unadjusted** |  |  |  |  |
| 51-55dBA | Ref | Ref | Ref | Ref |
| 56-60dBA | 1.08 (0.79, 1.47) | 1.08 (0.79, 1.47) | 1.62 (0.98, 2.68) | 2.00 (1.21, 3.32) |
| 61-65dBA | 0.86 (0.68, 1.09) | 0.86 (0.68, 1.09) | 1.05 (0.66, 1.68) | 0.84 (0.51, 1.37) |
| 66-70dBA | 1.23 (0.90, 1.67) | 1.23 (0.90, 1.67) | 1.26 (0.67, 2.37) | 1.23 (0.65, 2.33) |
|  |  |  |  |  |
| **Adjusted** |  |  |  |  |
| 51-55dBA | Ref | Ref | Ref | Ref |
| 56-60dBA | 0.95 (0.70, 1.29) | 0.95 (0.70, 1.29) | 1.54 (0.91, 2.59) | 1.82 (1.07, 3.07) |
| 61-65dBA | 0.85 (0.67, 1.08) | 0.85 (0.67, 1.08) | 1.14 (0.71, 1.85) | 0.82 (0.49, 1.37) |
| 66-70dBA | 1.26 (0.92, 1.73) | 1.26 (0.92, 1.73) | 1.37 (0.72, 2.61) | 1.31 (0.68, 2.52) |
